# High content quantitative imaging of *Mycobacterium tuberculosis* responses to acidic microenvironments within human macrophages

**DOI:** 10.1002/2211-5463.13537

**Published:** 2023-01-10

**Authors:** Beren Aylan, Laure Botella, Maximiliano G. Gutierrez, Pierre Santucci

**Affiliations:** ^1^ Host‐Pathogen Interactions in Tuberculosis Laboratory The Francis Crick Institute London UK; ^2^ Present address: Aix‐Marseille Univ, CNRS, LISM, IMM FR3479 Marseille France

**Keywords:** bacterial reporters, endolysosomes, high‐content fluorescence microscopy, human‐induced pluripotent stem cell‐derived macrophages, pH sensing and homeostasis, Tuberculosis

## Abstract

Intracellular pathogens such as *Mycobacterium tuberculosis* (Mtb) have evolved diverse strategies to counteract macrophage defence mechanisms including phagolysosomal biogenesis. Within macrophages, Mtb initially resides inside membrane‐bound phagosomes that interact with lysosomes and become acidified. The ability of Mtb to control and subvert the fusion between phagosomes and lysosomes plays a key role in the pathogenesis of tuberculosis. Therefore, understanding how pathogens interact with the endolysosomal network and cope with intracellular acidification is important to better understand the disease. Here, we describe in detail the use of fluorescence microscopy‐based approaches to investigate Mtb responses to acidic environments *in cellulo*. We report high‐content imaging modalities to probe Mtb sensing of external pH or visualise in real‐time Mtb intrabacterial pH within infected human macrophages. We discuss various methodologies with step‐by‐step analyses that enable robust image‐based quantifications. Finally, we highlight the advantages and limitations of these different approaches and discuss potential alternatives that can be applied to further investigate Mtb–host cell interactions. These methods can be adapted to study host–pathogen interactions in different biological systems and experimental settings. Altogether, these approaches represent a valuable tool to further broaden our understanding of the cellular and molecular mechanisms underlying intracellular pathogen survival.

## Graphical abstract

*Mycobacterium tuberculosis* is a successful pathogen that is able to subvert host‐defence mechanisms, including intracellular acidification. In this Research Protocol, we describe high‐content fluorescence microscopy approaches and step‐by‐step image‐based quantitative analysis to monitor and assess *Mycobacterium tuberculosis* responses to acidic environments within infected human macrophages.

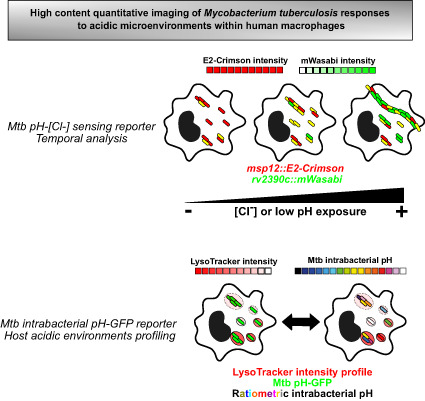

**Abbreviations**
EBs: embryonic bodies
iPSC: induced pluripotent stem cells
iPSDM: induced pluripotent stem cell‐derived macrophages
MDM: human monocyte‐derived macrophages
MOI: multiplicity of infection
Mtb: *Mycobacterium tuberculosis*
OD_600_
: optical density at λ_600nm_
TB: tuberculosis
v‐ATPase: vacuolar‐type ATPase

In macrophages, after uptake by phagocytosis, intracellular pathogens are directed to the endolysosomal pathway to be degraded. However, successful pathogens have evolved strategies to overcome these defence mechanisms. *Mycobacterium tuberculosis* (Mtb), the aetiologic agent of tuberculosis (TB), is one of them.

Compelling evidence shows that Mtb resides within heterogenous subcellular niches with distinct biochemical properties during infection [1]. During infection of host cells, Mtb uses multiple factors to manipulate phagosome maturation and prevent phagosome acidification [1, 2, 3, 4]. Mtb infection is associated with impaired acquisition of the vacuolar‐type ATPase (v‐ATPase) at the phagosome, thereby significantly reducing phagosome acidification [5]. The Mtb‐secreted protein tyrosine phosphatase PtpA binds to the subunit H of the human v‐ATPase and blocks its trafficking to the phagosome and subsequent phagosome acidification [6, 7]. In addition, numerous Mtb lipids, glycolipids or terpene nucleosides have also been identified as modulators of phagosome maturation and acidification [8, 9, 10, 11]. Mtb can also damage the phagosomal membrane to reach the nutrient‐rich cytosol of host cells, thereby avoiding the low pH environment of the phagosome. This process is mediated by the cooperation of the ESX‐1 Type VII secretion system and the phthiocerol dimycocerosate lipids [12, 13, 14, 15, 16, 17, 18]. Moreover, Mtb can endure acidic conditions within *in vitro* and *in cellulo* model systems, suggesting that Mtb can survive, adapt and replicate in the phagolysosome [19, 20, 21].

Several imaging modalities have allowed researchers to visualise and quantify phagosome maturation and acidification in real time [22]. For example, live‐cell imaging of Mtb fluorescent reporter strains combined with lysosomotropic and proteolytic probes showed that the interplay between Mtb and the endocytic pathway is dynamic in both mouse and human macrophages [23, 24, 25]. However, how intracellular microenvironments impact bacterial fitness and replication is still poorly understood. Moreover, recent studies have shown that the host cell activation status, nature of environments and subcellular localisation of Mtb impact antibiotic efficacy [26, 27, 28]. Altogether, these findings highlight the need of continuous development of unbiased high‐content imaging approaches that considers the intracellular and dynamic heterogeneity of Mtb localisation.

Here, we describe the use of a human macrophage model based on human‐induced pluripotent stem cell‐derived macrophages (iPSDM) combined with fluorescence microscopy‐based approaches, experimental design and analytical workflow to monitor Mtb sensing of host‐microenvironmental pH and Mtb intrabacterial pH homeostasis *in cellulo* [17, 27, 28]. We provide a step‐by‐step protocol enabling sample preparation, image acquisition and analysis. Finally, we discuss potential applications of imaging to study host–pathogen interactions together with the advantages and the current limitations of this approach.

## Materials

### *Mycobacterium tuberculosis* reporter strains

**Fig. 1:**
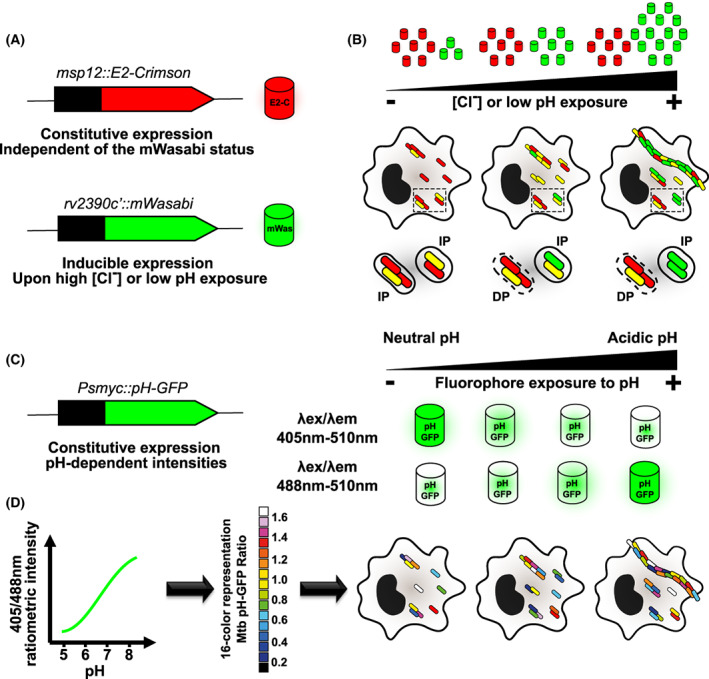
Schematic representation and principle of Mtb pH‐[Cl^−^] sensing and Mtb pH‐GFP reporter strains. (A) Schematic representation of the dual‐reporter strategy used to generate Mtb pH‐[Cl^−^] sensing reporter. The mWasabi‐encoding gene was cloned under the inducible promoter of *rv2390* (green signal), and the E2‐Crimson‐encoding gene was cloned under the strong constitutive promoter *msp12* (far‐red signal). (B) Principle of the Mtb pH‐[Cl^−^] sensing reporter within infected cells. Exposure and sensing of low pH and [Cl^−^] overtime is correlated with higher mWasabi levels, thereby increasing mWasabi/E2‐Crimson ratios as arbitrarily represented. A schematic representation of cells infected with the Mtb pH‐[Cl^−^] sensing reporter is shown. Zoom‐ins corresponding to dotted‐line boxes illustrate how subcellular localisation and host environments impact pH exposure, sensing and response below each micrograph. Mtb strains restrained within intact phagosomes (IP) display higher mWasabi/E2‐Crimson ratios overtime in contrast to Mtb strains that are able to damage phagosomes (DP) and reach the pH‐neutral cytosol therefore producing less mWasabi proteins (B. Aylan, E.M. Bernard, E. Pellegrino, L. Botella, A. Fearns, N. Athanasiadi, C. Bussi, P. Santucci, & M.G. Gutierrez, unpublished). (C) Schematic representation of the dual ratiometric strategy used to monitor intrabacterial pH homeostasis with the Mtb pH‐GFP reporter. The pH‐sensitive GFP‐encoding gene was cloned under the strong constitutive promoter *smyc* (dual green signal). The biological principle of the ratiometric pH‐GFP fluorophore is highlighted on the right panel. The two excitation/emission channels are inversely responding to pH exposure, thereby displaying distinct fluorescence intensity profiles which can be monitored noninvasively overtime. (D) Representation of ratiometric signal quantification and principle of the Mtb pH‐GFP reporter within infected cells. Fluorescence intensity ratio can be performed by dividing the fluorescence intensity acquired with excitation/emission channels of 405/510 nm by the one obtained at 488/510 nm *in vitro*. An increase in the 405/488 nm ratio reflects neutralisation or alkalinisation of the fluorophore environment, in our case Mtb intrabacterial pH. Inversely, a decrease in the 405/488 nm ratio highlights the acidification of the bacterial cytosol. The pH‐GFP intensity ratio can be shown as 16‐colour palette ranging from 0 to 1.6 units, which reflects the heterogeneity in mycobacterial pH homeostasis overtime as arbitrarily represented.

- *Mycobacterium tuberculosis* (Mtb) H37Rv strain (obtained from Douglas B. Young, The Francis Crick Institute, London, UK) was used in this study.
- Mtb pH‐[Cl^−^] sensing dual reporter (Fig. 1A,B) was obtained by transforming Mtb with the *rv2390c′::mWasabi‐msp12::E2‐Crimson* construct (B. Aylan, E.M. Bernard, E. Pellegrino, L. Botella, A. Fearns, N. Athanasiadi, C. Bussi, P. Santucci, & M.G. Gutierrez, unpublished), a derived version of *rv2390c′::GFP‐smyc′::mCherry* reporter strain [29], generated from plasmids pTEC15 and pTEC19 (kindly provided by Prof. Lalita Ramakrishnan, Addgene, Watertown, MA, USA; Plasmid #30174 and Plasmid #30178, respectively).
- Mtb pH‐GFP reporter strain (Fig. 1C,D) was obtained by transforming Mtb with the pUV15‐pH‐GFP construct (a kind gift from Prof. Sabine Ehrt, Addgene Plasmid #70045).
- *Complete Middlebrook 7H9 broth* consisting of Middlebrook 7H9 base (Sigma‐Aldrich, Gillingham, UK; Cat#M0178) supplemented with 0.2% glycerol (v/v) (Fisher Chemical, Loughborough, UK; Cat#G/0650/17), 0.05% Tween‐80 (v/v) (Sigma‐Aldrich, Cat#P1754) and 10% ADC (v/v) (BD Biosciences, Wokingham, Berkshire, UK; Cat#212352) was used to grow Mtb strains.
- Hygromycin B 50 μg·mL^−1^ (Invitrogen, Waltham, MA, USA; Cat#10687010), was used as appropriate selection marker when required.

### Human‐induced pluripotent stem cell culture and human‐induced pluripotent stem cell‐derived macrophages preparation

- KOLF2 iPSC cells (HPSI0114i‐kolf_2 iPSC, Public Health England Culture Collections, Cat #77650100) were used in this study.
- Vitronectin XF (Stem Cell Technologies, Cambridge, UK; Cat#100‐0763) coated dishes (Thermo‐Scientific, Waltham, MA, USA; 11309283).
- TrypLE™ (Life Technologies, Waltham, MA, USA; Cat#12604013).
- Essential 8™ Medium (Gibco, Waltham, MA, USA; Cat#A1517001).
- DPBS buffer (Gibco, Cat#14190‐144).
- AggreWell™ 800 plates (Stem Cell Technologies, Cat#34815).
- Anti‐Adherence Rinsing Solution (Stem Cell Technologies, Cat#07010).
- Y‐27632 ROCK inhibitor (Stem Cell Technologies, Cat#72307).
- hM‐CSF (Peprotech, London, UK; Cat#300‐25).
- hIL‐3 (Peprotech, Cat#200‐03).
- hBMP4 (Peprotech, Cat#120‐05).
- hVEGF (Peprotech, Cat#100‐20).
- hSCF (Peprotech, Cat#300‐07).
- 40 μm cell strainer (Corning, Glendale, AZ, USA; Cat#352340).
- *Factory complete medium* consisting of X‐VIVO15 (Lonza, Slough, UK; Cat#BEBP02‐061Q) containing 1% (v/v) Glutamax (Gibco, Cat#35050061), 0.1% (v/v) β‐mercaptoethanol (Gibco, Cat#21985023), 100 ng/mL hM‐CSF (Peprotech, Cat#300‐25) and 25 ng/mL hIL‐3 (Peprotech, Cat#200‐03).
- T225 EasyFlask (ThermoFisher, Cat#159934).
- 15‐cm dishes (Star Labs, Blakelands, UK; Cat#CC7672‐3614).
- *iPSDM differentiation complete medium* consisting of X‐VIVO15 (Lonza, Cat#BEBP02‐061Q) containing 1% (v/v) Glutamax (Gibco, Cat#35050061) and 100 ng/mL hM‐CSF (Peprotech, Cat#300‐25).
- Trypan blue staining (Sigma‐Aldrich, Cat#T8154).
- Automated cell counter (BioRad, Hercules, CA, USA; TC20™ Automated Cell Counter).

### Imaging and quantitative analysis of M. tuberculosis response to acidic microenvironments in cellulo

- Versene solution (Gibco, Cat#15040066).
- Cell scrapers (Sarsted, Leicester, UK; Cat#83.1830).
- Viewplates 96‐well glass bottom (PerkinElmer, Rodgau, Germany; 6005430) or
- Olefin‐bottomed 96‐well plate (PerkinElmer, 6055302).
- 12 mm or 22 mm aperture glass bottom dishes (WillCo‐dish®, Amsterdam, the Netherlands; Cat#GWST‐3512‐3522).
- Autoclaved glass beads 2.5–3.5 mm (VWR Chemicals, Lutterworth, UK; Cat#332124G).
- *iPSDM complete medium* consisting of X‐VIVO15 (Lonza, Cat#BEBP02‐061Q) containing 1% (v/v) Glutamax (Gibco, Cat#35050061).
- NH_4_Cl (Sigma‐Aldrich, Cat#A9434) quenching solution at 50 mM in 1× DPBS pH 7.2.
- DAPI (Invitrogen, Cat#D1306) staining solution in 1× DPBS (1 : 10 000).
- LysoTracker™ Red DND‐99 (Invitrogen, Cat#L7528) staining solution in *iPSDM complete medium* (1 : 5000).
- Opera Phenix high‐content imaging system (PerkinElmer) equipped with a 63× 1.15NA water immersion objective.
- Harmony 4.9 software (PerkinElmer).

## Methods

### A. Preparation of embryonic bodies and monocyte‐producing factories

N.B. The following steps require the use of a class II biosafety cabinet, the strict respect of conventional cell culture aseptic techniques and standard operating procedures to minimise contamination.

#### iPSC culture

1. Maintain iPSC in Vitronectin XF coated plates with Essential 8™ Medium.
2. Once the confluency reaches 70%, wash the cells one time with 1× DPBS and add 3 mL of Versene solution.
3. Incubate the cells at 37 °C for 5 min.
4. Remove the Versene solution gently and resuspend the cells within Essential 8™ Medium.
5. Plate the cells in 1 : 6 ratio.
6. Once iPSCs reach the required quantity, proceed to embryonic bodies (EBs) preparation.

#### EBs preparation and maintenance

7 Prior to EBs seeding, equilibrate an AggreWell™ plate with anti‐adherence rinsing solution.
8 Add 0.5 mL anti‐adherence rinsing solution to the wells and centrifuge at 3000 ***g*** for 3 min to remove any bubbles.
9 Aspirate and rinse the wells with 1 mL 1× DPBS.
10 Add 1 mL of E8 medium supplemented with 1 mm Y‐27632 ROCK inhibitor, 50 ng·mL^−1^ BMP‐4, 20 ng·mL^−1^ SCF and 50 ng·mL^−1^ VEGF to each well.
11 Centrifuge plate at 3000 ***g*** for 3 min and place the plate in CO_2_ incubator at 37 °C.
12 Remove medium from iPSC‐containing plate and rinse once with 1× DPBS.
13 Aspirate DPBS and add 1 mL room temperature TrypLE™ and incubate at 37 °C for 5 min.
14 Assess the cell detachment under microscope, if not incubate further for an additional 2–5 min.
15 Dilute the TrypLE™ solution containing the cells with 1× DPBS (1 : 10) and collect in a centrifuge tube.
16 Ensure that the cells are in single‐cell suspension by performing gentle up‐and‐down pipetting before centrifugation.
17 Centrifuge at 400 *g* for 5 min.
18 After centrifugation, remove the DPBS‐TrypLE™ solution and resuspend the cells in Essential 8™ Medium supplemented with 1 mm Y‐27632 ROCK inhibitor.
19 Ensure a final cell concentration of 4.0 × 10^6^/mL.
20 Add 1 mL of the cell suspension to AggreWell™ (making a total volume of 2 mL).
21 Centrifuge the plate at 100 ***g*** for 3 min.
22 Examine the cell distribution after the centrifugation and return the plate to CO_2_ incubator at 37 °C.
23 Change the medium daily by removing twice 50% of Essential 8™ Medium supplemented with 50 ng·mL^−1^ hBMP4, 50 ng·mL^−1^ hVEGF and 20 ng·mL^−1^ hSCF for an additional 3 days.

#### Factory preparation, macrophage differentiation and seeding

24 On day 4, harvest the EBs by flushing out of the well with gentle pipetting and filtering through an inverted 40 μm cell strainer.
25 Seed the EBs from each Aggrewell™ into one T225 flask in *Factory complete medium*.
26 Feed the monocyte factories once per week with 20 mL of *Factory complete medium* for 5 weeks until adequate number of monocytes can be observed in the supernatant.
27 Weekly harvest 20–40 mL of the supernatant and subsequently feed the factories with 20–40 mL with *Factory complete medium*.
28 Centrifuge the supernatant at 300 ***g*** for 5 min.
29 Resuspend the monocytes in *iPSDM differentiation complete medium*.
30 Plate 4–5 × 10^6^ cells per 10‐cm petri dish or 10–12 × 10^6^ cells per 15‐cm petri dish to differentiate over 7 days.
31 On day 4 after monocytes plating, replace 50% of the media with *iPSDM differentiation complete medium*.
32 After 7 days of differentiation, iPSDM are ready for harvesting and seeding.
33 Wash one time with 1× DPBS.
34 Add 3 mL of Versene solution to 10‐cm petri dish or 5‐mL to 15‐cm petri dish.
35 Incubate at 37 °C for 15 min.
36 Dilute the Versene solution with 1× DPBS (1 : 3) and gently scrape the macrophages.
37 Centrifuge the suspension at 300 ***g*** and check the viability of the macrophages by resuspending in *iPSDM complete medium*.

Note: At this stage, iPSDM can be collected and further characterised by flow cytometry for surface expression of conventional macrophage markers such CD14 and CD11b.

### B. Mtb growth and IPSDM infection

N.B. The following steps might require the use of a dedicated and appropriate laboratory. We recommend strictly respecting the requirements and standard operating procedures according to each biosafety‐level containment and in the case of Mtb manipulation the biosafety‐level 3 requirements.

1. Grow Mtb pH‐[Cl^−^] sensing reporter or Mtb pH‐GFP in 10 mL 7H9 complete medium in 50‐mL conical tubes to midexponential phase (OD_600nm_ ~ 0.5–1) under constant rotation at 37 °C.
2. The day before infection, seed the iPSDM at a density of 50 000 cells per well of a 96‐well plate or 300 000 macrophage per single WillCo‐dish.
3. Centrifuge the bacterial culture at 2000 ***g*** for 5 min and wash twice in 1× DPBS.
4. Shake the bacterial pellet vigorously for 1 min with 2.5–3.5 mm glass beads.
5. Resuspend the bacteria in 10 mL *iPSDM complete medium* and centrifuge at 300 ***g*** for 5 min to remove large clumps.
6. Recover the top 5–7 mL of bacterial suspension and measure the OD_600_.
7. Dilute the bacterial suspension to MOI:1, assuming OD_600_ of 1 is 1 × 10^8^ bacteria·mL^−1^ and gently add the inoculum to the cells.
8. After 2 h of uptake, remove the extracellular bacteria with two washes in 1× DPBS.
9. Add *iPSDM complete medium* and incubate the cells for the required time points at 37 °C and 5% CO_2_.

### C. High‐content fluorescence‐based quantitative imaging of Mtb response to pH within infected human iPSDM

N.B. The following steps might require the use of a dedicated and appropriate laboratory. We recommend strictly respecting the requirements and standard operating procedures according to each biosafety‐level containment and in the case of Mtb manipulation the biosafety‐level 3 requirements.

#### Chemical fixation, staining procedure and plate preparation for microscopy acquisition

**Fig. 2:**
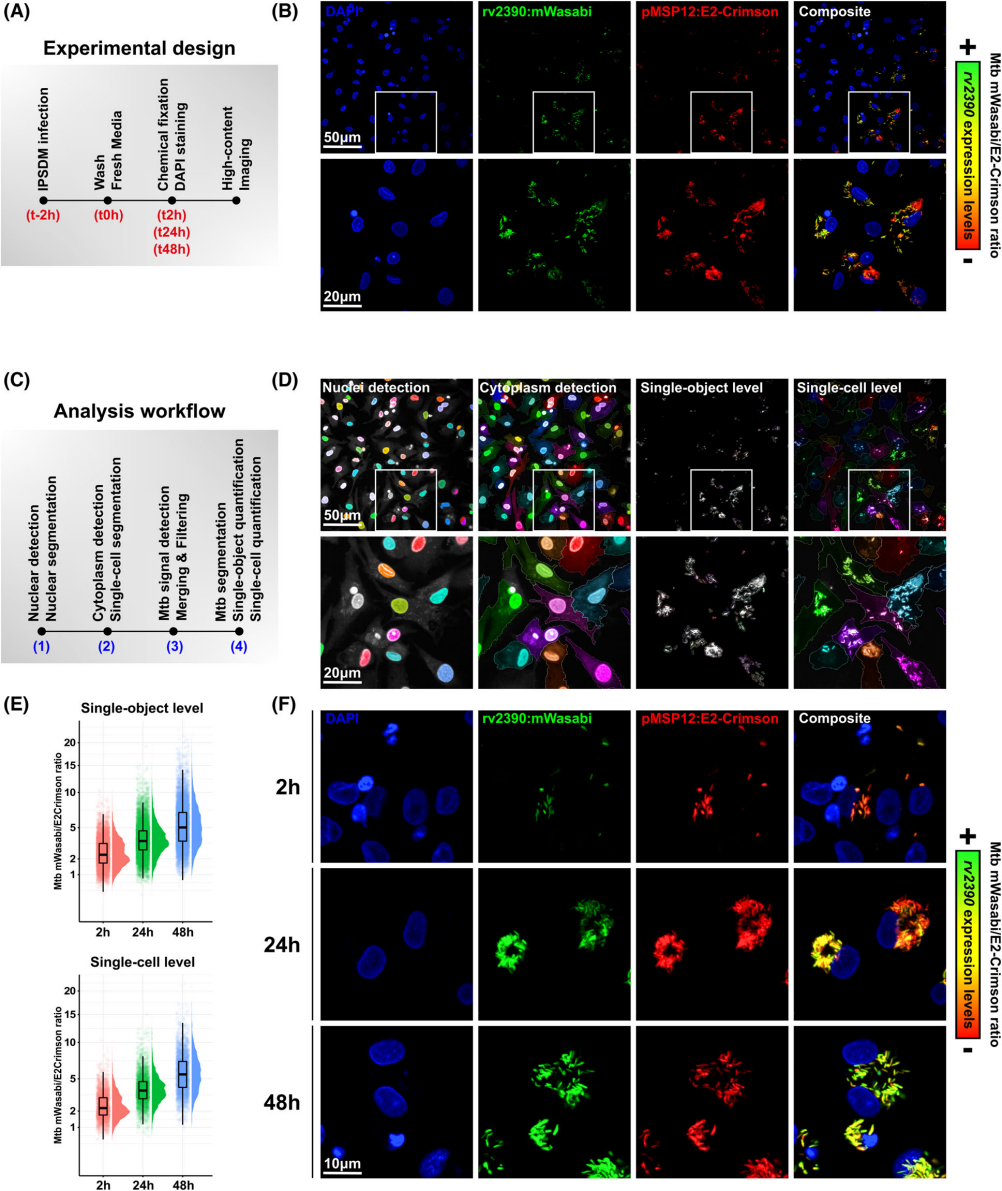
High‐content fluorescence‐based quantitative imaging of Mtb response to Cl^−^ and pH within infected human iPSDM. (A) Schematic representation of the experimental design followed to monitor Mtb response to intracellular Cl^−^ and pH. (B) Representative micrographs of Mtb pH‐[Cl^−^] sensing reporter‐infected iPSDM display DAPI labelling (blue), Mtb rv2390:mWasabi (green), Mtb pMSP12:E2‐Crimson (red) and the composite channel. Sensing of pH‐[Cl^−^] is correlated with higher mWasabi/E2‐Crimson ratios as arbitrarily represented with the relative scale associated with the right panel. Zoom‐ins are displayed below each micrograph. Scale bars correspond to 50 and 20 μm, respectively. (C) Schematic representation of the analytical workflow followed to quantitatively assess Mtb response to intracellular Cl^−^ and pH. (D) Representative micrographs of the segmentation and analysis pipeline routinely used to perform quantitative analysis of Mtb adaptation within the intracellular environment of IPSDM using the Mtb pH‐[Cl^−^] sensing reporter. Single‐cell segmentation is achieved by combining the ‘Find nuclei’ and ‘Find cytoplasm’ building blocks. Signal from both the mWasabi and the E2‐Crimson channels was detected using the ‘Find Image Region’ or alternatively the ‘Find Spot’ building block where a manual threshold was applied to accurately perform bacterial segmentation. mWasabi/E2‐Crimson ratio was determined by quantifying the mean of mWasabi and E2‐Crimson signals per single‐Mtb object or per single infected iPSDM. Scale bars correspond to 50 μm and 20 μm, respectively. (E) Quantification of Mtb mWasabi/E2‐Crimson ratio (510/633 nm) within infected iPSDM at *t*_2 h_, *t*_24 h_ and *t*_48 h_ postinfection. Top panel shows results obtained at the single object level, whereas bottom panel shows results obtained by performing single‐cell analysis. Results are displayed as raincloud plots where black boxplots are overlaid on top of individual raw data and associated with their respective density plots. (F) Representative micrographs of Mtb pH‐[Cl^−^] sensing reporter‐infected iPSDM at *t*_2 h_, *t*_24 h_ and *t*_48 h_ postinfection. Panels display DAPI labelling (blue), Mtb rv2390:mWasabi (green), Mtb pMSP12:E2‐Crimson (red) and the composite channel. Sensing of pH‐[Cl^−^] is correlated with higher mWasabi/E2‐Crimson ratios as arbitrarily represented with the relative scale associated with the right panel. Scale bar corresponds to 10 μm, respectively.

Note: Mtb pH‐[Cl‐] sensing reporter is based on two‐genetically encoded compatible fluorophores, mWasabi and E2‐Crimson. The expression of E2‐Crimson is placed under the strong and constitutive msp12 promotor and therefore is produced continuously at constant levels. On the contrary, the rv2390c*′*::mWasabi transcriptional fusion is responsible for the production of a bright and strong fluorescent signal upon exposure to low pH and high intracellular concentration of chloride. With this transcriptional fusion involving a stable fluorophore, it is important to consider that a delay in the sensing, signal emission and fluorophore degradation might happen. To detect, quantify and assess this signal with a higher temporal resolution, the use of destabilised or unstable fluorophores might be more appropriate.

1. At the required time postinfection (*e.g. t*_2 h_, *t*_24 h_, *t*_48 h_), collect the appropriate plate containing Mtb pH‐[Cl^−^] sensing reporter‐infected iPSDM (Fig. 2A).

2 Remove the *iPSDM complete medium* gently and wash one time with 1× DPBS.
3 Fix the plates overnight with a solution of 4% paraformaldehyde in 1× DPBS.

Note: After this step, plates can be removed from biosafety‐level‐3 laboratory following the appropriate standard operating procedures

4 Remove fixative solution and add 100 μL of 50 mm NH_4_Cl in 1× DPBS pH 7.2 quenching solution per 96‐well and ~ 500‐1000 μL in WillCo‐dishes.

Note: Fixative solution is highly toxic and must be removed under a chemical fume hood.

Note: A solution of 0.1 m glycine in 1× DPBS pH 7.2 could also be used as an alternative quenching solution

5 Incubate 10 min at room temperature.
6 Remove NH_4_Cl quenching solution and add DAPI staining solution in the same amounts as described in *Step C.4*.
7 Incubate 10 min in the dark at room temperature.
8 Remove DAPI staining solution and wash once with 1× DPBS.
9 Clean the bottom of the plate or WillCo‐dishes with wipes.

#### Imaging with the Opera Phenix system

10 Place carefully the plate inside the OPERA Phenix high‐content microscope.
11 Select the 63× 1.15NA water immersion objective.
12 Define the appropriate wells and fields of view that need to be acquired.

Note: We recommend repeating the acquisition on multiple fields of view to increase the number of infected cells imaged and set the overlap function at 5–10% in between fields. Setting the overlap function at 5–10% facilitates the generation of high‐quality global images that are less likely to be truncated by avoiding the loss of few pixels on the edge of each field upon acquisition.

13 To minimise resolution reduction, use the confocal mode, a binning of 1 and the default autofocus function.
14 Perform DAPI signal detection using at wavelengths (λex/λem) 405 nm/450 nm (Fig. 2B).
15 Perform Mtb pH‐[Cl^−^] sensing reporter signal using a sequential acquisition at wavelengths (λex/λem) 488 nm/500–550 nm for mWasabi and wavelengths (λex/λem) 595 nm/633–683 nm for E2‐Crimson (Fig. 2B).
16 Set the appropriate laser power and exposure time for all channels to obtain the best signal‐to‐noise ratio. Usually, power is set between 5% and 30% with an exposure time of 100 and 400 ms.
17 Set the acquisition to image each channel independently and use a minimum of 3–4 distinct focal z‐planes spaced with 0.5–1 μm for high‐resolution acquisition.
18 Perform automated acquisition, data collection, saving and relocation for further analysis.

#### Unbiased detection, segmentation and analysis using the harmony software (PerkinElmer, version 4.9)

19 Display the acquired images as a global image and assess the overall quality of the dataset for each well and each sample before establishing the analysis workflow (Fig. 2C).
20 Perform iPSDM detection by combining the ‘Find nuclei’ and ‘Find cytoplasm’ building blocks (Fig. 2D). With these tools host cell nuclei, cytoplasm, edges and background levels can be easily identified by using in‐built methods or manual thresholding based on fluorescent intensities. Combining appropriately the building blocks allows to obtain single‐cell segmentation (Fig. 2D). Assess the strength of the applied parameters and the quality of the single‐cell segmentation by selecting random fields of view in distinct wells. The generated mask should allow to discriminate individual cells that can be later subdivided as two main subpopulations, infected and noninfected cells.
21 Detect the signals from both the mWasabi and the E2‐Crimson channels, respectively, using the ‘Find Image Region’ or alternatively the ‘Find Spot’ building block where a manual threshold was applied to accurately define bacterial objects.
22 Merge the signal from the mWasabi and the E2‐Crimson channels by using the ‘Calculate Image’ and the function ‘By Formula’ by applying a channel A + B operation.
23 Signal‐to‐noise ratio can be improved by using the ‘Filter Image’ building blocks and a sliding parabola function. Such parameters should allow to perform an improved bacterial segmentation, and the generated Mtb mask can be now used to identify infected cells and quantify Mtb mean fluorescent signal per single object and/or per single IPSDM that was infected (Fig. 2D).
24 Determine mWasabi and E2‐Crimson ratio by quantifying the mean of mWasabi over E2‐Crimson fluorescence intensity per single‐Mtb object or per single iPSDM (Fig. 2E,F).
25 It is possible to include in the workflow the analysis of any additional parameters such as area, number or mean intensity that might be relevant. This can be done for any features of interest such as nuclei, infected and uninfected cells and bacterial objects.
26 Load the appropriate assay layout and perform the automated analysis.
27 Export the results as .csv files and perform data analysis using R studio or any other data visualisation software that can display violin plots, boxplots, scatterplots, density plots or raincloud plots (Fig. 2E,F).

Note: Individual raw fluorescence images can also be exported as TIFF files and alternatively displayed with open‐source software such as fiji/imagej software (https://imagej.net/software/fiji/) [30]. Step‐by‐step procedures similar to the one described previously can be done to perform quantitative analysis. Such workflow can be eventually fully automated using macro language and programming.

### D. High‐content fluorescence‐based quantitative imaging of Mtb intrabacterial pH homeostasis within infected human iPSDM

N.B. The following steps might require the use of a dedicated and appropriate laboratory. We recommend strictly respecting the requirements and standard operating procedures according to each biosafety‐level containment and in the case of Mtb manipulation the biosafety‐level 3 requirements.

#### Staining procedure and plate preparation for microscopy acquisition

**Fig. 3:**
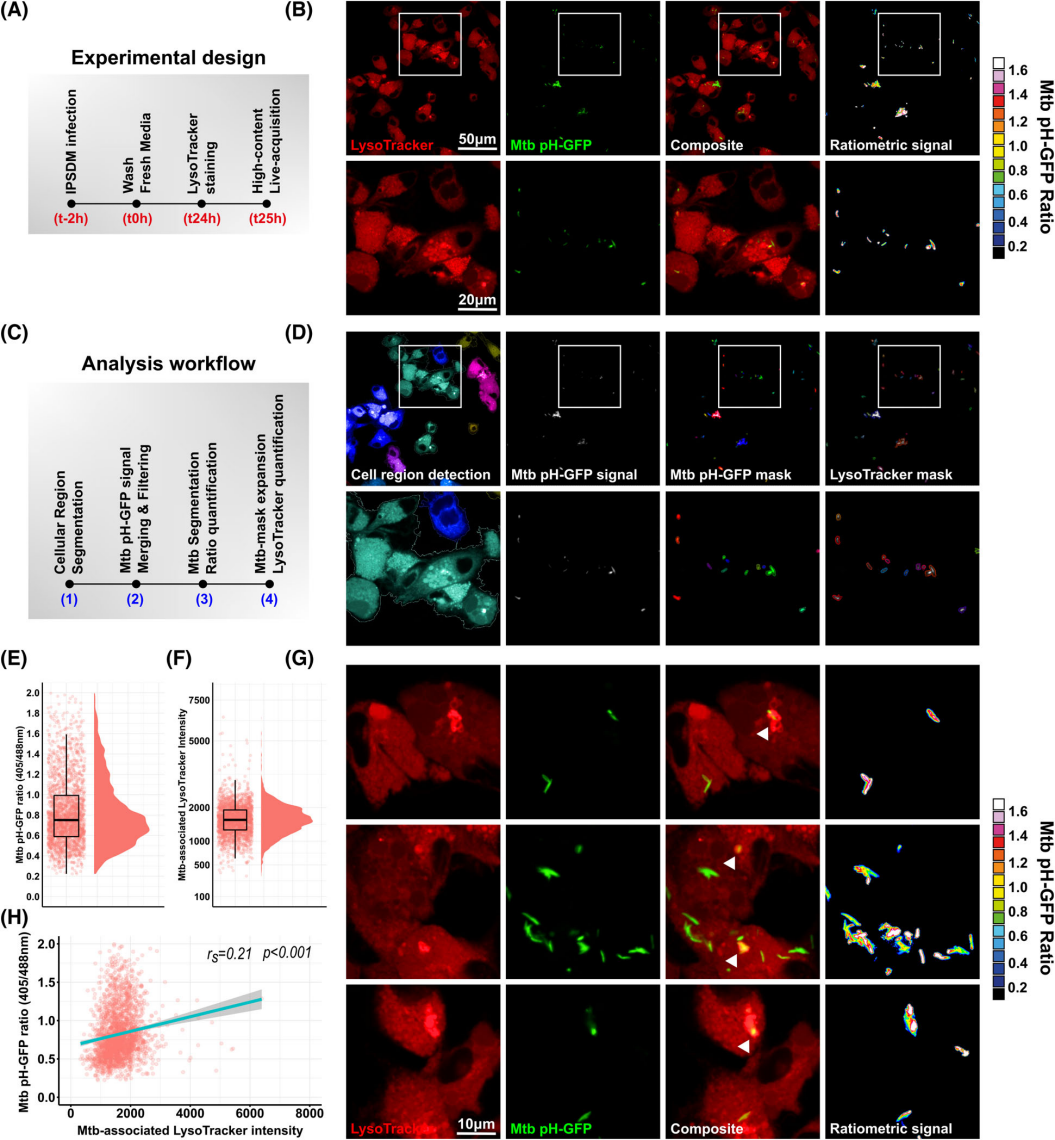
High‐content fluorescence‐based quantitative imaging of Mtb intrabacterial pH homeostasis within infected human iPSDM. (A) Schematic representation of the experimental design followed to monitor Mtb intrabacterial pH within infected iPSDM. (B) Representative micrographs of Mtb pH‐GFP reporter‐infected iPSDM with LysoTracker™‐Red labelling. Panels display LysoTracker labelling (red), Mtb pH‐GFP (green), the composite channel and the pH‐GFP ratiometric signal displayed as a 16‐colour palette ranging from 0 to 1.6 units. Ratiometric signal was obtained by dividing the fluorescence intensity acquired with excitation/emission channels of 405/510 nm by the one obtained at 488/510 nm. Zoom‐ins are displayed below each micrograph. Scale bars correspond to 50 and 20 μm, respectively. (C) Schematic representation of the analytical workflow followed to quantitatively assess Mtb intrabacterial pH within infected IPSDM. (D) Representative micrographs of the segmentation and analysis pipeline routinely used to perform quantitative analysis of Mtb intrabacterial pH within the intracellular environment of IPSDM using the Mtb pH‐GFP reporter. Cellular region was detected based on the fluorescent signal of the red emission channel using the ‘Find Image Region’ building block and the ‘Absolute Threshold’ function. Intracellular Mtb pH‐GFP was detected based on the GFP signal obtained into both λex 405 nm/λem 500–550 nm and λex 488 nm/λem 500–550 nm channels using the ‘Find Image Region’ building block and the ‘Absolute Threshold’ function. Signals from the two GFP channels were merged and filtered using the ‘Calculate Image’ and ‘Filter Image’ building blocks. This Mtb mask was used to quantify Mtb pH‐GFP mean fluorescent signal per single object from both 405‐nm/510‐nm and 488‐nm/510‐nm channels. Ratiometric signals were obtained by dividing the mean intensity quantified λex 405 nm/λex 488 nm for each object. To quantify Mtb‐associated LysoTracker™ intensity, the Mtb mask was slightly extended using the ‘Find Surrounding Region’ building block using method A with an individual threshold value of 0.8 and conservation of the input region. (E) Quantification of Mtb pH‐GFP ratio (405/488 nm) within infected iPSDM and (F) Quantification of Mtb‐associated LysoTracker™ mean fluorescence intensities within infected iPSDM. Results are displayed as raincloud plots where black boxplots are overlaid on top of individual raw data and associated with their respective density plots. (G) Representative micrographs display LysoTracker™ labelling (red) and Mtb pH‐GFP (green). Ratiometric signal was obtained by dividing the fluorescence intensity acquired with excitation/emission channels of 405/510 nm by the one obtained at 488/510 nm. Arrows indicate LysoTracker™‐positive Mtb. Ratiometric signal is displayed as a 16‐colour palette ranging from 0 to 1.6 units. Scale bar corresponds to 10 μm. (H) Spearman's correlation between Mtb‐associated LysoTracker™ (*x* axis) and Mtb pH‐GFP ratio (405/488 nm) (*y* axis) signals in individual bacterial region of interests within infected iPSDM. The cyan line shows the linear regression model; the Spearman rank correlation coefficient (*r*_*s*_) and the corresponding *P* value were calculated by using the ggplot R package and two‐tailed statistical *t* test.

Note: Chemical fixation of the Mtb pH‐GFP reporter strain interferes with the assessment of intrabacterial pH. Paraformaldehyde treatment fixes the fluorophore in a state where it displays homogeneous pH ratios that approximate the pH value of the fixative solution. For detection, quantification and assessment of intrabacterial pH, it is required to perform live‐cell imaging.

1. At the required time postinfection (*t*_24 h_), collect the appropriate plate containing Mtb pH‐GFP reporter‐infected iPSDM (Fig. 3A).

2 Remove the *iPSDM complete medium* gently and wash one time with 1× DPBS.
3 Add 100 μL of *iPSDM complete medium* containing LysoTracker™ Red DND‐99 staining solution (1 : 5000) and incubate for 30–45 min at 37 °C and 5% CO_2_.

Note: LysoTracker™ Red DND‐99 is a fixable dye that retains its fluorescent properties after aldehyde fixation; however, for optimal detection, quantification and biological relevance, we recommend avoiding chemical fixation and perform live‐cell imaging.

4 Replace the staining solution with *iPSDM complete medium* and seal the plate according to the standard operating procedures for live‐cell microscopy acquisition in biosafety‐level 3 laboratory.
5 Clean the bottom of the plate or WillCo‐dishes with wipes.

#### Microscopy acquisition with the Opera Phenix high‐content screening platform

6 Place carefully the plate within the microscope with TCO settings at 37 °C and 5% CO_2_.

Note: We recommend setting the TCO settings 10–15 min before loading the plate within the microscope

7 Select the microscope settings by repeating *Steps C.11 to C.13*.
8 Mtb pH‐GFP signal was detected using a sequential acquisition at excitation wavelengths (λex) 405 and 488 nm, and an emission wavelength (λem) comprises between 500 and 550 nm (Fig. 3B).
9 LysoTracker™ signal was detected using excitation wavelength (λex) 561 nm, and (λem) an emission wavelength comprises between 570 and 630 nm (Fig. 3B).
10 Adjust the microscope settings and perform acquisition by repeating *Steps C.16 to C.18*.

#### Unbiased detection, segmentation and analysis using the harmony software (PerkinElmer, version 4.9)

11 Display the acquired images as a global image and assess the overall quality of the dataset for each well and each sample before establishing the analysis workflow (Fig. 3C).
12 Perform iPSDM detection with the LysoTracker™ signal by using the ‘Find Image Region’ building block and the ‘Absolute Threshold’ function. With this function and the use of an appropriate manual thresholding, this should allow to obtain a robust discrimination between cellular region and acellular background areas (Fig. 3D).
13 Detect the GFP fluorescent signal obtained into both channels (λex/λem) 405 nm/500–550 nm and (λex/λem) 488 nm/500–550 nm using the ‘Find Image Region’ building block and the ‘Absolute Threshold’ function or alternatively the ‘Find Spot’ building block where a manual threshold was applied to accurately define bacterial objects.
14 Merge the signal from the two GFP channels by using the ‘Calculate Image’ building block and the function ‘By Formula’ by applying a channel A + B operation (Fig. 3D)
15 If required, improve signal‐to‐noise ratio by using the ‘Filter Image’ building blocks and a sliding parabola function.
16 Perform mask expansion by using the ‘Find Surrounding Region’ building block. (Fig. 3D).

Note: In the Harmony software and the ‘Find Surrounding Region’ building block, we recommend to use the method A with an individual threshold value of 0.6–1 and conservation of the input region, which seemed to be the more appropriate with our experimental datasets. Mask expansion is not essential if only the determination of Mtb pH‐GFP ratio is required. However, this step is required to generate one single mask that can be now used to quantify Mtb mean pH‐GFP ratiometric signal per single object within infected cells and its respective LysoTracker™‐associated fluorescence mean intensity.

17 Determine Mtb pH‐GFP ratio by quantifying the mean of GFP intensities per single‐Mtb object in both channels (λex/λem) 405 nm/500–550 nm and (λex/λem) 488 nm/500–550 nm. Ratiometric signals can be obtained by dividing the mean intensity quantified at (λex/λem) 405 nm/500–550 nm by the one obtained at (λex/λem) 488 nm/500–550 nm for each object. (Fig. 3E,G).

Note: This ratiometric analysis allows obtaining a relative signal reflecting intrabacterial pH variation at the single object level. To obtain the corresponding pH values, a calibration curve must be performed. Briefly, mycobacterial cells expressing the pH‐GFP reporter are collected and resuspended in phosphate citrate buffer adjusted from pH 5 to pH 8.5. Cells are lysed by mechanical disruption, and protein concentration of each lysate is quantified. Protein concentration of bacterial lysates is normalised and further uses to determine GFP intensities in both channels (λex/λem) 405 nm/500–550 nm and (λex/λem) 488 nm/500–550 nm using a spectrofluorometer. Fluorescence ratios are then fitted to pH values with the sigmoidal Hill equation to obtain the appropriate calibration curve.

18 Determine the respective Mtb‐associated Lysotracker™ fluorescence mean intensity per each single‐Mtb object (Fig. 3F,G).
19 Load the appropriate assay layout and perform the automated analysis.
20 Export the results as .csv files and perform data analysis using R studio or any other data visualisation software that can display violin plots, boxplots, scatterplots, density plots or raincloud plots (Fig. 3E,F,H).

### Tips and tricks

#### Advantages and limitations of iPSDM to study cell biology of infection

The recent development of iPSC‐based models has provided a very powerful tool to produce specific human immune cell types that can be used to study the molecular and cellular bases of host–pathogen interactions [31]. Being able to establish cellular models from healthy and patient donors and/or perform genetic engineering onto these cells represents an advantage to study human disease and particularly the human macrophage biology of infection [31]. These stem cell‐based models, such as human iPSDM, with physiologically relevant biological features, are a powerful alternative to immortalised cell lines [31]. However, iPSC culture, differentiation and characterisation can be time‐consuming and work intensive with a high financial cost when compared to conventional cell lines, bone marrow‐derived macrophages or human monocyte‐derived macrophages. Several protocols have been previously published for macrophages, allowing to perform iPSC and iPSDM culture using commercial and noncommercial media for production after an extensive characterisation of the cellular phenotypes [32, 33, 34, 35, 36, 37].

The experimental design proposed within this protocol can be adapted to alternative cellular models. This requires optimisation of key steps such as infection, cell seeding density, MOI, bacterial uptake, kinetics (*e.g*. slow‐ *vs*. fast‐growing pathogens), staining procedures and detection/analysis workflow.

#### Advantages and limitations of this experimental protocol and analysis workflow

Depending on the model, experiments in the context of host–pathogen interactions cannot be performed with fixed samples and require live‐cell imaging to monitor the spatiotemporal dynamics of intracellular events with high resolution. In this protocol, we provide two distinct methodologies: the first one probes Mtb transcriptional response towards low pH/high chloride to better define the biochemical nature of intracellular environments (*section C*, Fig. 2), and the second one monitors the host subcellular acidification profile and Mtb intrabacterial pH in real‐time (*section D*, Fig. 3). It is also important to highlight that the first one can be performed with chemically fixed samples (*section C*, Fig. 2) whereas the latter is only compatible for live‐cell imaging (*section D*, Fig. 3).

Before defining whether to perform live‐ or fixed‐cell imaging, it is essential to consider various aspects related to the nature of the biological questions. Dynamic processes might often require real‐time imaging with spatial and temporal resolution and the tracking of individual cells and specific events.

We suggest to carefully consider the biological and/or biophysical features of the reporters and probes available as chemical fixation can alter their properties and function and live‐cell probes can be toxic, altering critical cellular processes. Some dyes/probes are sensitive to photobleaching, whereas others might require strong and repeated illumination cycle which may result in phototoxicity. All these parameters will inform on whether live‐cell imaging or fixed fluorescence imaging is more appropriate/feasible.

The protocol described in *section C* (Fig. 2) has also been successfully applied in real‐time in our laboratory and therefore can be used to answer different types of biological questions under different experimental conditions. On the contrary, the second protocol described in *section D* (Fig. 3) can only be performed in live condition, as the pH‐GFP reporter is not suitable for chemical fixation [20, 28, 38].

Live imaging of Mtb pH‐GFP requires a dual excitation/emission step, which includes illumination with a diode 405 nm laser. This laser can induce strong phototoxic effects when performed in long‐term live‐cell imaging. We recommend assessing these parameters before the experiment, including the effects on cell viability after incubation with dyes/probes or exposure to multiple laser pulses. Carefully adjusting the microscope settings such as laser power and exposure time might prevent the phototoxicity. For long‐term live‐cell imaging, it is important to minimise light exposure by increasing the time between frames or opt for compromising resolution by reducing scanning time. However, an insufficient number of pixels leads to sampling artefacts, and therefore, such compromise must be done only if a low spatiotemporal resolution is sufficient to address the biological question. In addition to phototoxicity, photobleaching might also occur with some probes or fluorophores and similar procedures as above can be used to maximise signal‐to‐noise ratio and reduce bleaching overtime. Alternatively, during analysis, a photobleaching algorithm‐based correction can be applied, if minimal, with some specific software or plugins [23].

The multiple acquisitions and analysis workflows presented in this work were performed using a spinning disk‐based Opera Phenix high‐content microscope and the harmony software (PerkinElmer, version 4.9). However, it can be adapted to alternative acquisition with other single‐point confocal light scanning microscopes, using open‐source software. Indeed, similar experimental procedures have been described [17, 24, 25, 28] with confocal light scanning microscope where the data were analysed using the open‐source fiji/imagej software (https://imagej.net/software/fiji/) [30].

#### The quest to achieve single‐bacterium resolution and segmentation

The protocol we recommend is well suited to detect single bacterial events within a single cell. It nevertheless has reduced power to achieve constant single‐Mtb segmentation. Our method focusses on objects and does not exclude these objects containing more than one bacterium. This is mainly due to the atypical mycobacterial division process as nonuniform heterogenous sticky rods [39, 40, 41]. Softwares that have been developed to individually segment bacteria such as *E. coli* or *B. subtilis* [42, 43, 44, 45, 46, 47, 48] are unfortunately not suitable to achieve automated single‐Mtb segmentation accurately. In addition, most of these algorithm‐based segmentation tools are commonly used to detect and segment bacteria at one specific focal point in two dimensions and do not allow to perform segmentation using three‐dimensional fluorescence microscopy time‐lapse images.

These limitations could be addressed by combining of super resolution microscopy with machine‐learning approaches that could achieve automated analysis of single mycobacterial cells in 3D overtime. Such approaches have been used recently to accurately segment and monitor single bacterial behaviour within 3D biofilms [49, 50]. These tools will revolutionise automated segmentation and analysis to achieve single‐Mtb‐organelle segmentation and will provide new insights on the molecular and cellular mechanisms underlying the cell biology of TB infection.

## Conflict of interest

The authors declare no conflict of interest.

## Authors contribution

BA, LB, MGG and PS conceived the study and designed the experiments. BA, LB and PS performed the experiments. MGG secured funding and contributed to reagents and analytic tools. BA, MGG and PS provided intellectual input by analysing and/or discussing data. BA, MGG and PS edited and wrote the manuscript. All authors read and approved the final manuscript before its submission.

## Data Availability

The data that support the findings of this study are available from the corresponding authors max.g@crick.ac.uk or psantucci@imm.cnrs.fr upon reasonable request.
